# Multi-site microbiota alteration is a hallmark of kidney stone formation

**DOI:** 10.1186/s40168-023-01703-x

**Published:** 2023-11-25

**Authors:** Kait F. Al, Benjamin R. Joris, Brendan A. Daisley, John A. Chmiel, Jennifer Bjazevic, Gregor Reid, Gregory B. Gloor, John D. Denstedt, Hassan Razvi, Jeremy P. Burton

**Affiliations:** 1 Centre for Human Microbiome and Probiotic Research, Lawson Health Research Institute, London, ON Canada; 2 Department of Microbiology and Immunology, The University of Western Ontario, London, ON Canada; 3 Department of Biochemistry, The University of Western Ontario, London, ON Canada; 4 Molecular and Cellular Biology Department, University of Guelph, Guelph, ON Canada; 5 Division of Urology, Department of Surgery, The University of Western Ontario, London, ON Canada

**Keywords:** Microbiota, Kidney stones, Gut microbiota, Urinary microbiota, Shotgun metagenomic sequencing, Urology

## Abstract

**Background:**

Inquiry of microbiota involvement in kidney stone disease (KSD) has largely focussed on potential oxalate handling abilities by gut bacteria and the increased association with antibiotic exposure. By systematically comparing the gut, urinary, and oral microbiota of 83 stone formers (SF) and 30 healthy controls (HC), we provide a unified assessment of the bacterial contribution to KSD.

**Results:**

Amplicon and shotgun metagenomic sequencing approaches were consistent in identifying multi-site microbiota disturbances in SF relative to HC. Biomarker taxa, reduced taxonomic and functional diversity, functional replacement of core bioenergetic pathways with virulence-associated gene markers, and community network collapse defined SF, but differences between cohorts did not extend to oxalate metabolism.

**Conclusions:**

We conclude that multi-site microbiota alteration is a hallmark of SF, and KSD treatment should consider microbial functional restoration and the avoidance of aberrant modulators such as poor diet and antibiotics where applicable to prevent stone recurrence.

Video Abstract

**Supplementary Information:**

The online version contains supplementary material available at 10.1186/s40168-023-01703-x.

## Background

Kidney stone disease, or nephrolithiasis, is a prevalent condition that causes significant morbidity to sufferers and is a draining financial burden to healthcare systems [1]. Although traditionally considered to be an affliction of obese and middle-aged men, prevalence has risen in recent decades, specifically in young women and children [2–5]. The human microbiota is known for its role in systemic health and disease, including metabolic syndrome, cardiovascular disease, and diabetes [6–8]. All of these conditions are comorbidities associated with nephrolithiasis, and their increasing prevalence over recent years along with that of stone disease indicate systemic declines in our population’s overall health. Importantly, the human microbiota is also implicated in nephrolithiasis, but a consensus on the mechanisms behind this relationship remains elusive.

Calcium oxalate (CaOx) is the most common crystalline composition of stones, followed by calcium phosphate, uric acid, struvite, and cystine [9]. Oxalate is a toxic terminal metabolite produced endogenously, while a smaller portion is consumed in the diet [10]. *Oxalobacter formigenes* (now also *O. aliiformigenes*, *O. paeniformigenes*, and *O. paraformigenes*) [11] was thought to be a key modulator of oxalate in the human body — it utilizes the molecule as its sole carbon source, and some studies have shown that intestinal colonization with this bacterium may be associated with lower urinary oxalate levels and subsequently a lower risk of developing CaOx stones [12, 13]. However, other members of the gut microbiota are also capable of degrading oxalate [14, 15], and many recent studies have found no difference in colonization rates of *O. formigenes* between healthy persons and stone formers [16–21]. Thus, it remains unclear if direct oxalate metabolism by gut colonizers is the key to preventing kidney stones.

Beyond oxalate utilization, previous studies have demonstrated generalized “dysbiosis” in the intestinal microbiota of kidney stone formers [16–18, 20–26]. However, the significant perturbations determined in these studies are seldom consistent; this may be an artifact of small sample size, or different sequencing and analysis methodologies. Most of the studies to date have also focused primarily on the presence or absence of *O. formigenes* and direct oxalate utilization pathways, but the narrow focus towards these analyses may be overemphasizing their true functional significance to the disease pathology.

Except for infection, the urinary system was historically believed to be sterile; however, a urinary microbiota in healthy humans has been well described in the last decade [27, 28]. This discovery has led investigators to question the role microbes may play in nephrolithiasis [29]. Indeed, while struvite stones are known to be associated with urinary tract infections (UTI), recent culture-dependent and culture-independent studies have also confirmed the presence of bacteria in calcium-based stones [16, 30, 31]. Recent evidence suggests that direct bacterial oxalate utilization could also play a role in the urinary tract [32], but much is still unknown about how resident bacteria may be contributing to the disease, and whether stone-bound microbes result from an aberrant urinary or gut microbiota. Which body site is of most significance to the pathology is still unclear, and an untargeted holistic role for the microbiome beyond direct oxalate utilization has been underexplored.

The aim of the present study was to comprehensively characterize the urinary and gut microbiota of kidney stone formers (SF) and healthy controls (HC) to assess body site-specific microbial contributions to nephrolithiasis. The distant site of the oral cavity was also explored due to its known associations with systemic disease [33]. It was anticipated that this would provide insights into the role of bacteria from multiple body sites in stone formation, and foundational knowledge upon which personalized medicine and targeted therapies could be developed for the prevention and treatment of nephrolithiasis.

## Methods

### Patients and sample collection

Eighty-three active nephrolithiasis patients (SF) were recruited from the Urology Department at St. Joseph’s Hospital in London, Ontario, along with thirty healthy control participants (HC) between August of 2015 and January of 2019. Ethical approval for the KiSMi study was granted by Lawson Health Research Institute (CRIC R 15–117) and the Health Sciences Research Ethics Board at the University of Western Ontario (REB #105443) in London, Ontario. Written consent was obtained from all the study participants at the time of study inclusion and the methods were carried out in accordance with the approved guidelines. Inclusion and exclusion criteria for the participants are provided in Supplementary Table 1. Briefly, neither HC nor SF could have an active gastrointestinal infection or antibiotic exposure within the 30 days prior to study enrollment (although in actuality all participants had at least 90 days without antibiotic exposure, an acceptable criterion based on current literature [34]). Additionally, HC could not have inflammatory bowel disease, a history of gastric bypass surgery, a history of urosepsis in the past year, a urinary diversion, an indwelling catheter, or intermittent catheterization performed. In comparison, the SF were still eligible to be included with these health issues, but only three SF had IBD (which was accounted for in multivariate analyses, Supplementary Data), and the other conditions were not present in any SF. All SF patients who met the inclusion criteria were recruited during regularly scheduled clinic appointments prior to their ureteroscopy (URS) or percutaneous nephrolithotomy (PCNL) for stone removal; HC subjects were approached in the community, and age and sex matched to SF with a loose frequency-matching approach [35].

### Sample processing and DNA extraction

Upon recruitment, participants were asked about relevant demographic and medical history including antibiotic usage and their history of urinary tract infections (Table 1, Supplementary Data 1A). Following enrolment, HC were evaluated by ultrasound to confirm stone-free status. All participants then provided a midstream urine sample, oral saliva swab, and 102 out of 113 mailed in a fecal sample on toilet paper [36]. They also completed a validated food frequency questionnaire based on estimated yearly intake and portion size to provide daily nutrient estimates [37]. During surgical stone removal, additional clinical samples were collected from SF where possible: urine, upon first insertion of the catheter and prior to instilling saline into the urinary tract, and stone fragments from PCNL. Prophylactic antibiotics were administered peri-operatively, differentiating the operating room (OR) urine and stone samples from all other specimens collected. In the majority of cases, patients undergoing PCNL had 5 days of oral ciprofloxacin pre-operatively, followed by IV ampicillin and gentamycin in the OR and then for 24 h after surgery. URS patients would typically receive IV cefazolin in the OR and were then discharged home with a 3–5-day course of trimethoprim/sulfamethoxazole or ciprofloxacin. These OR specimens were placed by the surgeon into a sterile collection cup. An OR environmental control sample was also collected where a sterile urine container containing 200 μL of nuclease-free water was left open beside the patient for the duration of their surgery. The composition of additional available stone fragments was analysed by the St. Joseph’s Healthcare Toxicology and Special Chemistry Lab by Fourier transform infrared spectrophotometry.

All urine samples (i.e. from HC as well as pre-operative and OR urine from SF) were processed in two portions within 2 h of collection. Where possible, 10 mL of whole urine was collected and frozen at − 80 °C for high-performance liquid chromatography (HPLC) analyses of urinary metabolites. The remainder was processed as previously described [38, 39] and stored for future 16S rRNA gene sequencing. Briefly, the entire remaining volume of urine was centrifuged for 10 min at 5000 × *g*, after which the supernatant was decanted off and the pellet was stored dry at − 80 °C until DNA extraction. If the total urine volume was under 25 mL, only 2 mL of whole urine was reserved for HPLC. The urine volume that resulted in the pellet for 16S rRNA gene sequencing was recorded to identify confounding factors in the downstream sequencing analysis associated with processing conditions.

Oral swabs were frozen at − 80 °C within 2 h of their initial collection and stored for future DNA extraction and 16S rRNA gene amplicon sequencing. In a validated method adapted from the American Gut Project [36, 40, 41], fecal samples were collected by participants at home and mailed to the laboratory, where they were frozen at − 80 °C within 2 h of their receipt. Within 2 h of collection, the water inside the OR environmental control sample was shaken in the cup for 2 min and the entire volume was transferred to a PCR-grade Eppendorf tube and frozen at − 80 °C.

Within 2 h of their initial collection, one stone fragment per patient was transferred to a PCR-grade Eppendorf tube and frozen at − 80 °C.

For DNA extraction, urine, stone, and OR environmental control samples were randomized across 96-well extraction plates together; oral and fecal samples were extracted on separate plates to mitigate potential contamination to the other samples which were of lower biomass in comparison.

Samples were thawed and processed in a sterile biosafety hood. Using tweezers sterilized with RNase AWAY™, the kidney stone was transferred to a sterile cell strainer mounted onto an empty 50-mL conical tube. New sterile cell strainers and conical tubes were used for every sample. Two milliliters of nuclease-free water was gently rinsed over the external surface of the stone. The stone was then transferred to a mortar and pestle that was sterilized with 5% sodium hypochlorite followed by RNase AWAY™ and pulverized into sand-like fragments. The fragments were suspended in 100 µL of nuclease-free water and pipetted directly into wells of the bead plate of the DNeasy PowerSoil HTP 96 Kit utilized for DNA extraction. Urine pellets were thawed and suspended in 100 μL of nuclease-free water, then pipetted into the bead plate. The 100 μL OR environmental control water sample was transferred directly to the bead plate. Oral swabs were cut directly into the wells of the bead plate with RNase AWAY™-treated scissors. Toilet paper samples were dissected and trimmed with RNase AWAY™-treated scissors and forceps such that a piece of visibly soiled paper approximately 1 cm^2^ in size was added directly to the bead plate.

Two wells in every plate were left empty and acted as negative controls. Two positive controls, or spikes, were added to each plate and were 100 μL of pure bacterial culture: Spike 1 was *Escherichia coli* strain DH5α, and Spike 2 was *Staphylococcus aureus* strain Newman. For preparation of the spikes, a single colony of the bacteria was inoculated into 10 mL of Luria–Bertani (LB) broth and grown overnight at 37 °C. One hundred-microlitre aliquots of the overnight cultures were portioned into 1.5-mL Eppendorf tubes and frozen at − 80 °C. For each DNA extraction plate, a single tube of both spikes was thawed and pipetted directly into the PowerSoil HTP bead plate with PCR-grade filter tips. DNA was isolated from samples using the DNeasy PowerSoil HTP 96 Kit according to the manufacturer’s instructions with minor adjustments as described previously [38]. DNA was stored at − 20 °C until PCR amplification.

### 16S rRNA gene sequencing of urine, stone and oral swab samples

PCR amplification was completed using the Earth Microbiome universal primers, 515F and 806R, which are specific for the V4 variable region of the 16S rRNA gene [42]. Primers and barcode sequences are listed in Supplementary Table 2. PCR reagent set-up was performed using a Biomek® 3000 Laboratory Automation Workstation (Beckman-Coulter, Mississauga, ON, CAN). Ten microlitres of each left- and right-barcoded primers (3.2 pMole/μL) was arrayed in 96-well plates such that each well contained a unique combination of left and right barcodes. Two microlitres of DNA template was added to the primer plate, followed by 20 μL of Promega GoTaq hot-start colourless master mix. The reaction was briefly mixed by pipetting, then plates were sealed with foil plate covers and centrifuged for 2 min at room temperature at 2250 × *g*.

Amplification was carried out using an Eppendorf thermal cycler (Eppendorf, Mississauga, ON, CAN), where the lid temperature was maintained at 104 °C. An initial warm-up of 95 °C for 4 min was utilized to activate the GoTaq, followed by 25 cycles of 1 min each of 95, 52, and 72 °C. Sequencing was carried out at the London Regional Genomics Centre (http://www.lrgc.ca; London, ON, CAN). Amplicons were quantified using pico green and pooled at equimolar concentrations before cleanup. Using the 600-cycle MiSeq Reagent Kit, paired-end sequencing was carried out as 2 × 260 cycles with the addition of 5% ɸX-174 at a cluster density of ~ 1100. Reads were exported as fastq files (uploaded to NCBI Sequence Read Archive, BioProject ID PRJNA856314 (urinary) and PRJNA856641 (oral). Reads were demultiplexed using Cutadapt [43], quality filtered utilizing the DADA2 pipeline [44], and assigned taxonomy with the SILVA (v138) training set [45]. Sequencing read counts are available in Supplementary Data 1Z. Samples and sequence variants (SVs) were pruned such that the final dataset utilized in downstream taxonomic analyses retained samples with > 1000 reads, SVs present at > 1% relative abundance in any sample, and SVs with > 10,000 total reads across all urinary samples. Due to the low-abundance nature of the samples, additional stringent assessment using the decontam R package [46] was performed to assess the presence of likely contaminant SVs, after which one additional SV was removed from the dataset. Inferencing of microbial metagenomes was performed with PICRUSt2 [47, 48].

### Whole shotgun metagenomic sequencing of stool samples

Fecal samples from 25 healthy control participants and 36 confirmed CaOx stone forming patients underwent shotgun metagenomic sequencing at The Centre for Applied Genomics at The Hospital for Sick Children in Toronto, ON, CAN. The SF samples were selected based on patients that had stone fragments extracted during surgery which were confirmed to be predominantly CaOx by Fourier transform infrared spectrophotometry. Samples from both cohorts were further pared to include only those surpassing DNA yield and quality thresholds: of the total 30 HC, 5 were excluded, and of the total 40 CaOx SF samples, 4 were excluded. DNA concentration was quantified using the Qubit™ dsDNA HS Assay Kit. Approximately 100 ng of DNA was PCR amplified and prepared for sequencing using the Nextera DNA Flex Library Prep Kit. The amplified library was purified and enriched for amplicons ~ 350 bp, then sequenced using an S1 Flowcell on the Illumina NovaSeq 6000.

Reads were exported as fastq files (BioProject ID PRJNA649273), quality assessed using FastQC [49], trimmed with Trimmomatic [50], and mapped against the human genome (Hg38) using Bowtie2 [51]. Sequencing read counts are available in Supplementary Data 1Z. Human reads were discarded, and the remaining unmapped reads were utilized in downstream analyses. The MGnify pipeline was used to annotate metagenomic functional potential [52]. Metagenomic bins were generated by mapping the reads from all samples to each raw assembly. MetaBat2 [53] was used to group contigs in the resultant alignment files based on their coverage and GC content. This process was repeated for sample-by-sample assemblies rather than a pooled assembly. Afterwards, all high-quality bins (assessed with CheckM [54]) were pooled together and dereplicated at 99% sequence identity using dRep [55]. Reads were then mapped back to the dereplicated set of genomes. PhyloPhlAn [56] was used to assign taxonomy to the bins, and counts were determined with Bowtie2. Taxonomic annotation was also performed with MetaPhlAn [57], which corroborated the PhyloPhlAn findings, but are not reported here. Functional annotation was also performed with the HUMAnN 3.0 pipeline [57], which generally corroborated the MGnify findings, but are not reported here.

### High-performance liquid chromatography

Urinary oxalate concentration was measured in parts per million with HPLC and normalized to creatinine [58–60]. Reserved whole urine samples were thawed and vortexed for 30 s. Using PCR-grade filter tips, 50 μL of urine was transferred to an Eppendorf tube for creatinine quantification, and 950 μL of urine was transferred to a 15-mL conical tube for oxalate quantification.

For quantifying creatinine, 450 μL HPLC H_2_O and 500 μL of HPLC acetonitrile were added to the urine and vortexed. The urine mixture was incubated at 4 °C for 15 min, facilitating precipitation of debris. Samples were then centrifuged for 15 min at 16,000 × *g* at 4 °C and filtered through 0.2-μm syringe filters into labelled amber HPLC vials (Agilent, Mississauga, ON, CAN). Standards of creatinine were prepared in HPLC H_2_O at concentrations of 1, 5, 10, 50, 100, 150, 200, 250, and 300 ppm. The Agilent 1100 HPLC was utilized with the conditions stated in Supplementary Table 3.

For oxalate quantification, the 950 μL of urine (or oxalic acid standards at concentrations of 1, 10, 25, 50, 100, 150, 200, 250 ppm) was combined with 50 μL of 10 M HCl and 1 mL of 0.1 M *o*-phenylenediamine dissolved in 4 M HCl and vortexed. The tubes were capped, being careful to tighten the lids as much as possible. The tubes were then incubated in a laboratory oven at 100 °C for 6–7 h, then moved to 4 °C overnight. The following day, volume in the tube was carefully inspected and tubes that had experienced cracking or evaporation were discarded, requiring repeat processing. Five hundred microlitres of 200 mM KHPO_4_ (pH 7.0) and 480 μL of 10 M KOH were added to the tubes with gentle vortexing. One millilitre of the mix was then transferred to labelled Eppendorf tubes and incubated at 4 °C for 15 min, then centrifuged for 15 min at 16,000 × *g* at 4 °C. Large pellets were present and were carefully avoided when transferring the entire volume to a 0.2-μm syringe filter; samples were filtered into labelled amber HPLC vials. The HPLC was utilized with the conditions stated in Supplementary Table 3.

### Statistical analysis

**Fig. 1:**
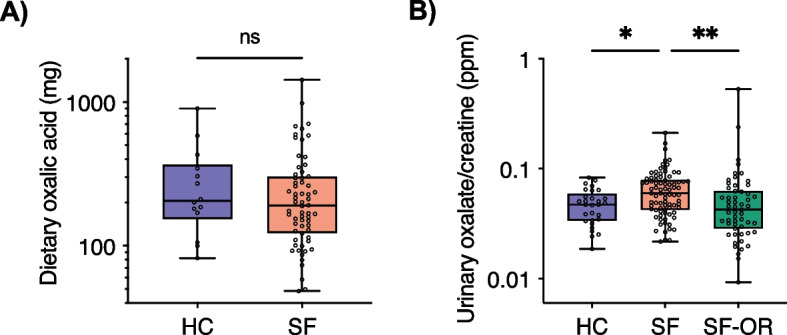
Urinary, but not dietary oxalate levels differ between healthy controls and stone formers. **A** The approximate daily value of oxalic acid as measured through a diet history questionnaire was comparable between patient groups. HC (*n* = 14), SF (*n* = 64). **B** Urinary oxalate concentrations were determined with HPLC and normalized to creatinine levels. SF pre-operative urine had the highest oxalate concentrations (Kruskall-Wallis test with Dunn’s multiple comparisons). Data represent the median (line in box), IQR (box), and minimum/maximum (whiskers); HC (*n* = 29), SF (*n* = 83), SF-OR (*n* = 55)

**Fig. 2:**
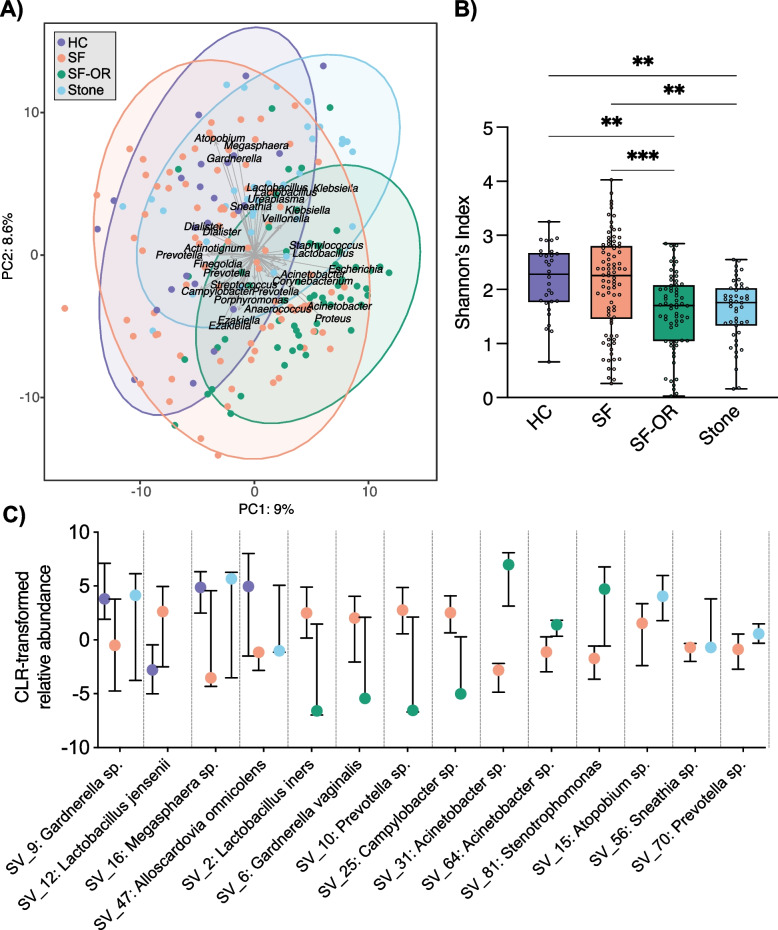
Compositional analysis of urine and stone microbiota. **A** PCA was performed on CLR-transformed Aitchison distances of all urine and stone samples. Each coloured point represents a sample. Distance between samples on the plot represents differences in microbial community composition, with 17.6% of total variance being explained by the first two components shown. Strength and association for genera are depicted by the length and direction of the grey arrows, respectively. Points are coloured by sample type and ellipses represent the 95% confidence intervals of sample types. Samples significantly differed by type and time (envfit *P* value < 0.05). **B** Shannon’s Index of alpha diversity was compared between sample groups. OR urine samples from stone patients had the lowest diversity (Kruskall-Wallis test with Dunn’s multiple comparisons, * *P* < 0.05, ** *P* < 0.01, *** *P* < 0.001). Data represent the median (line in box), IQR (box), and minimum/maximum (whiskers). **C** SVs were significantly distinct between stone former pre-operative urine compared to healthy control urine, stone former OR urine, and stones (Benjamini–Hochberg corrected Wilcoxon test *P* < 0.05, ANCOM W value > 0.7 threshold, ALDEx2 GLM effect size >|0.5|). Data represent the median and IQR; HC (*n* = 25), SF (*n* = 83), SF-OR (*n* = 59), Stone (*n* = 34)

**Fig. 3:**
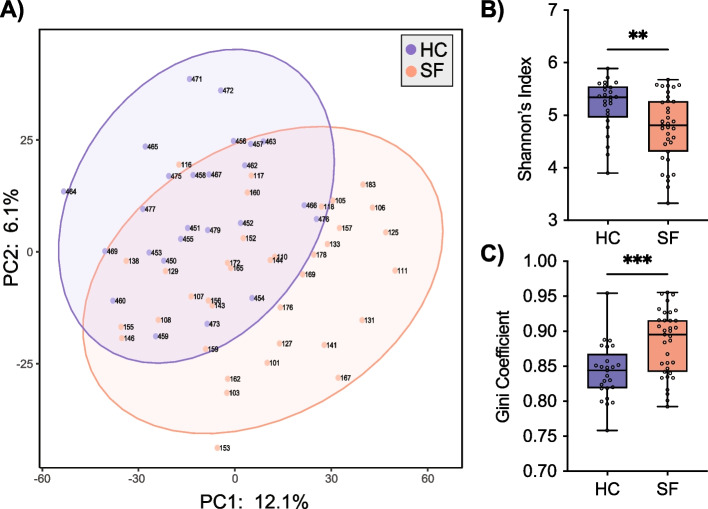
Stone-former gut microbiota differs from healthy controls. **A** PCA was performed on CLR-transformed Aitchison distances of metagenomic taxonomic bin assemblies from HC and SF fecal samples. Each coloured point represents a sample. Distance between samples on the plot represents differences in microbial community composition, with 18.2% of total variance being explained by the first two components shown. Points are coloured by sample type and ellipses represent the 95% confidence intervals of sample types. Samples significantly differed by cohort (envfit *P* value < 0.1). **B** Shannon’s Index of alpha diversity was significantly decreased in SF. **C** Gini coefficient of community inequality was significantly elevated in SF. Mann–Whitney tests, *P < 0.05, ***P < 0.001. Data represent the median, IQR, and minimum/maximum; HC (*n* = 25), SF (*n* = 36)

**Fig. 4:**
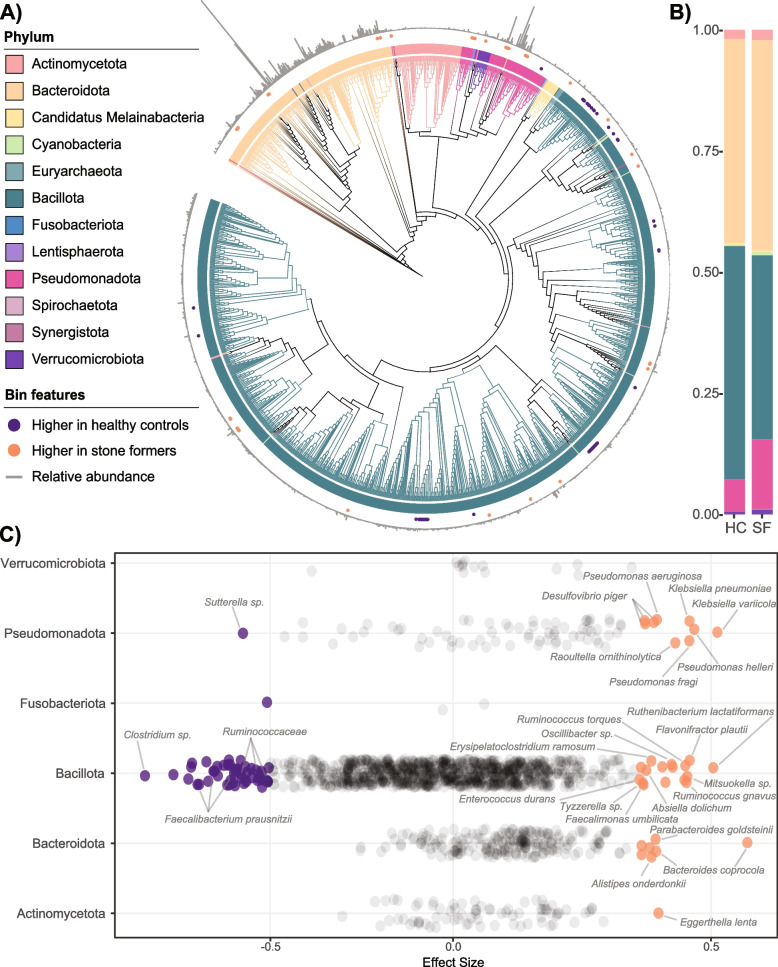
Phylogeny and differentially abundant gut microbiota taxa. **A** A maximum-likelihood phylogenetic tree of dereplicated genomes from the gut microbiota. The outermost grey bars represent the overall prevalence of the taxonomic bin. Orange and purple dots in the second layer denote taxonomic bins that were significantly more abundant in SF or HC, respectively (Benjamini–Hochberg corrected Wilcoxon test (*P* < 0.1) and effect size >|0.5|). Tree branches are coloured by phylum. **B** Average relative phylum abundance bar plot of HC and SF cohorts. Each vertical bar represents the average relative abundance within the cohort, coloured by phylum. **C** Effect sizes of taxa are coloured by cohort of enrichment and labelled where taxonomic information is available. Coloured species were significantly different by Benjamini–Hochberg corrected Wilcoxon test (*P* < 0.1) and effect size >|0.5|

**Fig. 5:**
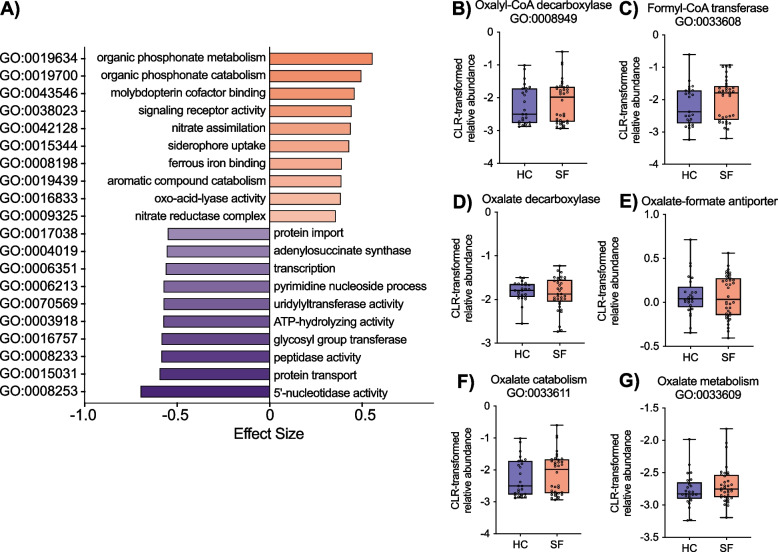
SF gut microbiota differs functionally from HC, but not in direct oxalate handling. **A** Effect size of the ten most differentially abundant gene ontology (GO) terms per cohort are coloured by cohort of enrichment. All GO terms shown were significantly different by Benjamini–Hochberg corrected Wilcoxon test (*P* < 0.1). **B–G** The relative abundance of oxalate handling genes was not different between cohorts by Bonferroni corrected Mann–Whitney *U* test. Data represent the median, IQR, and minimum/maximum; HC (*n* = 25), SF (*n* = 35)

**Fig. 6:**
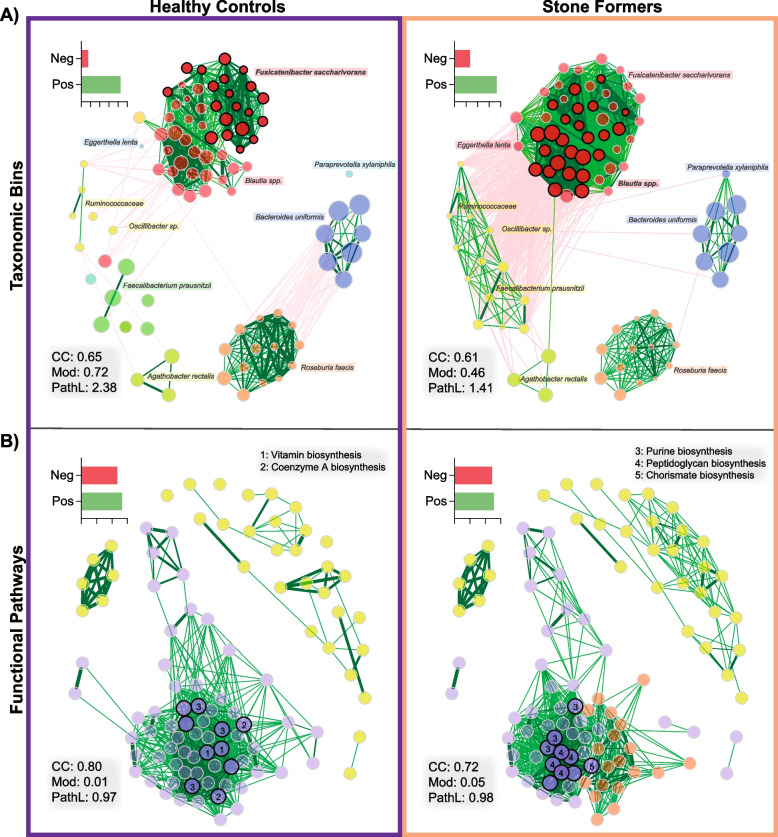
Co-occurrence networks demonstrate divergent community structures between HC and SF. Network inference was built upon 1000 bootstrap iterations of Pearson correlation coefficients from CLR-transformed taxonomic and functional pathway counts. **A** Nodes represent individual taxonomic bins, and clusters are labelled with corresponding species; the fifty nodes with the highest degree are displayed. **B** Nodes represent functional pathways; the eighty nodes with the highest degree are displayed. Nodes are coloured by clusters and sized by their CLR-transformed abundance, with nodes in bold representing hubs. Numbers within hubs correspond to the common functional pathways of interest. Edges with positive estimated interactions are coloured in green, and negative estimated interactions are coloured in red; percentage of edge positivity is displayed in the inset bar chart. CC = clustering coefficient, Mod = modularity, PathL = average path length

Sequencing data analysis was performed conservatively in agreement with best practices in the field [61], using CoDaSeq, zCompositions, ALDEx2, ANCOM2, Vegan, and core R packages [62–71]. Specifically, significant confounders were determined with the envfit function from the Vegan R package [64] and accounted for in multivariate analysis with generalized linear models (GLM) using ALDEx2 (Supplementary Data) [62]. Fixed effect formulae used for GLM comparisons are reported in the Supplementary Data. All reported *P* values are corrected for false discovery rate (FDR) where applicable. In the microbiota differential abundance tests, significance was considered for FDR-corrected *P* values *P* < 0.1 and/or effect size >|0.5|. For all other comparisons, *P* < 0.05 was considered significant. The phylogenetic tree of shotgun metagenomic bin taxonomic annotations was generated with PhyloPhlAn [56] and visualized with iTOL [72]. The selection of balances for microbial signatures (R package selbal) was used to determine significant microbial signatures predictive of kidney stone disease [73]. Co-occurrence network analyses were performed with the R package NetCoMi [74]. Per-cohort network inference was performed on 1000 bootstrap iterations of Pearson correlation coefficients from CLR-transformed taxonomic and functional pathway count tables. Sample numbers, statistical significance, and names of statistical tests with corresponding FDR corrections are provided in the main text and figure legends for Figs. 1, 2, 3, 4, 5 and 6, Supplementary Figs. 1–4, and the Supplementary Data. Shapiro–Wilk tests of normality and statistical tests on participant metadata and urinary oxalate were performed in GraphPad Prism (v8.3.1) and R.

## Results

### Kidney stone microbiome (KiSMi) cohort characteristics

The KiSMi cohort consisted of 113 participants, of which 83 were SF and 30 were HC (Supplementary Data 1A). This yielded 179 urine samples, 113 oral swabs, 47 stone fragments, and 102 fecal samples (Supplementary Fig. 1). HC were matched to SF based on age, sex, and most comorbidities (Supplementary Data), but differed in their history of UTI, smoking status, and antibiotic history. However, no participants had antibiotic exposure for at least 90 days prior to enrollment and initial sample collection (SF had prophylactic antibiotic exposure at the OR timepoint) (Table 1). SF were evenly divided between those who received percutaneous nephrolithotomy (PCNL) compared to ureteroscopic (URS) surgery. Of SF, 84% had recurrent kidney stones and 77% were CaOx stone formers. HC and SF participants did not significantly differ in their estimated dietary intake of oxalate (Fig. 1A), nor any of the other 216 measured features from the diet history questionnaire after correcting for false discovery rate; however, several trends were observed in decreased vitamin, saturated fatty acid, phytoestrogen, and other micronutrient levels (Supplementary Fig. 2, Supplementary Data 1B, C). The urinary oxalate/creatinine ratio was significantly higher in the SF patients at the pre-operative time point compared to HC and SF at the OR timepoint (Fig. 1B, Supplementary Data 1D).

**Table 1:** KiSMi study participants characteristics

**Metric**		**Healthy controls**	**Stone patients**	***P* value**
**No. enrolled**		30	83	
**Sex**		**n (%)**	**n (%)**	
	**Female**	11 (37)	32 (39)	
	**Male**	19 (63)	51 (61)	0.99^a^
**Age**		**M (SD)**	**M (SD)**	
		55 (11.1)	58 (11.5)	0.28^b^
**BMI**		**M (SD)**	**M (SD)**	
		27.6 (4.6)	30.2 (7.0)	0.08^c^
**Other health features**		**n (%)**	**n (%)**	
**History of stones**		0 (0)	70 (84)	<0.0001^a^
**History of UTI**		9 (30)	43 (52)	0.05^a^
**Smoking status**				
	**Current smoker**	0 (0)	12 (14)	
	**Non-smoker**	30 (100)	71 (86)	0.03^a^
**Comorbidities**	**Cardiac**	3 (10)	8 (10)	0.99^a^
	**Hypertension**	8 (27)	32 (39)	0.37^a^
	**Diabetes**	3 (10)	12 (14)	0.75^a^
	**Hypothyroidism**	3 (10)	9 (11)	0.99^a^
**Years since antibiotic use**		**M (SD)**	**M (SD)**	
		5.53 (5.8)	3.4 (4.0)	0.004^c^
**Stone removal procedure**			**n (%)**	
	**PCNL**		40 (48)	
	**Ureteroscopy**		43 (52)	
**Major stone component**				
	**Calcium oxalate**		40 (77)	
	**Calcium phosphate**		1 (2)	
	**Uric acid**		7 (13)	
	**Struvite**		3 (6)	
	**Cystine**		1(2)	
	**Unknown (not tested)**		31	

^a^Fisher’s exact test of categorical variables

^b^Unpaired two-tailed t-test for normally distributed continuous variables

^c^Mann-Whitney U test for non-normally distributed continuous variables

### 16S rRNA gene amplicon sequencing reveals distinct multi-site taxonomic and functional communities in SF

The most proportionally abundant genera in the urinary and stone dataset were *Escherichia* (29.7%), *Lactobacillus* (12.8%), *Staphylococcus* (10.3%), *Gardnerella* (7.3%), and *Streptococcus* (7.3%) (Supplementary Data 1E). The sequence counts were centred log ratio (CLR) transformed and a Euclidian distance was applied, generating sample-wise Aitchison distances, which were analysed with principal components analysis (PCA) [68]. The PCA biplot displays clustering of samples based on sample type, which was validated by *envfit* from the R package vegan (*P* < 0.05) (Fig. 2A, Supplementary Data 1F). Sample types differed by alpha diversity, whereby SF urine collected during stone removal surgery (OR urine) had the lowest Shannon’s index, and the HC and pre-operative SF urine samples had the highest (Fig. 2B, see Supplementary Data 1G-H for additional alpha diversity metrics). As sample type (stone vs. urine) and time (pre-operative vs. OR) were deemed significant drivers of microbiota variation (*envfit P* < 0.05), samples were investigated in separate comparisons on these bases to determine significantly altered taxa while adjusting for significant covariates (Supplementary Data 1F). The relative abundance of several SVs was discordant between the urine from HC and SF, SF and SF-OR, and SF urine and stones (Benjamini–Hochberg corrected Wilcoxon test with ALDEx2, and ALDEx2 GLMs; Fig. 2C, Supplementary Data 1I). Specifically, *Gardnerella* sp., *Megasphaera* sp., and *Alloscardovia omnicolens* were relatively more abundant in HC urine compared to SF urine, whereas *Lactobacillus jensenii* was relatively enriched in SF. Within stone patients, *Lactobacillus iners*, *G. vaginalis*, *Prevotella* sp., and *Campylobacter* sp. were relatively depleted at the OR timepoint (following antibiotic exposure and collected via catheter), while the nosocomial pathogens *Acinetobacter* spp. and *Stenotrophomonas* sp. were enriched. Compared to SF urine samples, stones harboured relatively more *Gardnerella* sp., *Megasphaera* sp., *A. omnicolens, Atopobium* sp., *Sneathia* sp., and *Prevotella* sp. Stones of calcium, struvite, uric acid, and cystine compositions harboured a microbiota surpassing the stringent filtering conditions implemented; however, potentially due to small sample sizes when sub-grouped by stone type, the microbiota was not driven by crystalline composition (*envfit P* > 0.05, Supplementary Data 1F and Supplementary Fig. 3).

Functional inferencing of the urinary samples revealed numerous genetic functions and pathways that were enriched in SF (pre-operative) relative to HC (Supplementary Data 1 J-K), including virulence-associated iron acquisition [75], enterobacterial common antigen biosynthesis [76], polmyxin resistance [77], stress-response ppGpp biosynthesis [78], and most significantly (standardized effect size 0.78) pyridoxine (vitamin B6) biosynthesis. Vitamin B6 is a cofactor for over 140 reactions in humans [79], including the detoxification of glyoxylate to glycine by the enzyme alanine-glyoxylate aminotransferase (AGT); deficiency of this vitamin and subsequent reduction in AGT activity can lead to increased urinary oxalate excretion [80, 81]. Thus, while systemic and intestinal vitamin B6 may have therapeutic benefits for stone formers, its role in the bladder is not well understood, and conversely has been anecdotally attributed to bladder sensitivity and incontinence symptoms [82, 83]. In contrast, urinary samples from SF were significantly depleted relative to HC in vitamin B12 biosynthesis, butyrate biosynthesis, and several basal bioenergetic enzymatic pathways.

The salivary microbiota from both cohorts was dominated by *Streptococcus* spp. (average abundance > 50%), followed by *Haemophilus*, *Gemella*, and *Escherichia* spp. (3–5%) (Supplementary Data 1L). While saliva samples from SF trended towards an increased relative abundance of the periodontal pathobionts *Porphyromonas endodontalis*, *Fusobacterium nucleatum*, and *Prevotella* sp., alongside depleted *Streptococcus* spp. compared to HC, this did not reach statistical significance after multiple testing correction and adjusting for significant covariates (Supplementary Data 1M), nor did differences based on functional inference testing (Supplementary Fig. 4, Supplementary Data 1N-P). However, the saliva samples from SF had significantly higher diversity than HC by multiple diversity metrics (Supplementary Data 1Q-R).

### Shotgun metagenomic sequencing reveals significant alterations in SF gut microbiota

A total of 102 fecal samples were collected from which DNA was extracted. Of these, 61 were selected for deep profiling with whole shotgun metagenomic sequencing. These comprised samples from 25 HC, and 36 confirmed CaOx SF, all with adequate DNA yield and quality for shotgun library preparation. Sample 153 was deemed to be an outlier via the CoDaSeq R package [67] and removed from downstream analyses (Fig. 3A). Samples separated by cohort based on PCA (Fig. 3A), and alpha diversity metrics (Fig. 3B, C). Specifically, Shannon’s index was significantly decreased in SF, and Gini coefficient of community inequality was significantly increased, illustrating that the SF harbour a gut microbiota with less diversity and more unequal distribution of taxa (Supplementary Data 1S-T).

Corroborating previous studies, *Faecalibacterium prausnitzii*, *Agathobacter rectalis*, *Bacteroide*s spp., *Roseburia* spp., *Alistipes* spp., *Ruminococcus* spp., and *Blautia* spp. were among the highest relative abundances across all gut samples (Fig. 4A, Supplementary Data 1U) [84, 85]. After correcting for significant covariates (Supplementary Data 1V), eighty-two bacterial taxa were differentially abundant between HC and SF, including relative enrichment of numerous pathobionts and disease-associated microbes in SF, with notable decreases in unclassified taxa from the phylum Bacillota and the health-associated *F. prausnitzii* (Fig. 4B, Supplementary Data 1W). *O. formigenes* abundance was not differentially abundant between cohorts, and similar to recent reports was only detected at very low abundance in two SF participants [21]. There were no significant differences between HC and SF in archaeal or eukaryotic taxa; however, a *Faecalibacterium* phage virus (*Taranisvirus*) was enriched in HC (effect size 0.53, Supplementary Data 1W).

The selection of balances (selbal) [73] was utilized to identify microbial signatures most predictive of stone disease. At the taxa level, the balance most associated with SF status was resolved through higher relative abundance of *Desulfovibrio piger* with respect to *Clostridium* sp. AM49-4BH and an unknown taxon from the phylum Bacillota (Supplementary Fig. 5A). At the genus level, the balance most associated with SF status was resolved through a higher relative abundance of *Pseudomonas* and *Proteus* with respect to an unknown genus from the phylum Fusobacteriota (Supplementary Fig. 5B).

Functionally, SF were significantly depleted in the relative abundance of numerous essential housekeeping functions (protein transport, transcription, bioenergetic machinery) and enriched in virulence and inflammatory processes (phosphonate metabolism [86], molybdopterin enzymatic processes [87], nitrate utilization [88], and iron acquisition [75]) (Fig. 5A, Supplementary Data 1X). In contrast, no differences were observed between cohorts in any of the genes associated with direct oxalate handling investigated here (Fig. 5B–G). Interestingly, bacterial uridylyltransferase activity was depleted in SF (Supplementary Data 1X), an enzyme that when deficient in humans can result in CaOx kidney stone formation via galactosemia [89].

### Network analysis reveals community-wide imbalance in SF microbiota

Co-occurrence networks were constructed for taxonomic and functional profiles of the gut microbiota to determine significant interactions (Fig. 6A, B). Both qualitatively and quantitatively, the taxa network was strikingly different between HC and SF cohorts (Fig. 6A, Supplementary Data 1Y), with significantly different network hubs (*P* = 0.028, all *Fusicatenibacter saccharivorans* in HC, and the majority *Blautia* spp. in SF), correlations, and overall structure (betweenness centrality *P* < 0.0001). Of note, the network hubs in SF were strongly negatively correlated with core health-associated gut microbes such as *F. prausnitzii* [90], and positively correlated with the inflammation-associated and SF-enriched *E. lenta* [91], while those correlation patterns were absent in HC. Network modularity, a marker of evolutionarily well-adapted community function [92], was also lower in SF (0.72 in HC vs. 0.46 in SF).

The network of interactions modelled on functional pathways also contrasted between cohorts (Fig. 6B), with significantly different network hubs (*P* = 0.032) and overall structure (betweenness centrality *P* < 0.0001). Hubs of folate, riboflavin, and coenzyme A biosynthesis were absent in SF, while peptidoglycan biosynthesis, and potentially virulence-associated hubs including purine [93, 94] and chorismate biosynthesis [95], were present. Of note, the overarching hub cluster with connection to major housekeeping functions such as glycolysis and coenzyme A biosynthesis was severed in SF. These network findings demonstrate that rather than a single or set of few microbes driving differences between the cohorts, there is a complete divergence of SF microbiome structure at a systems-level compared to HC.

## Discussion

This study demonstrated that independent of diet, the microbiome in stone formers is altered at multiple anatomical sites — evidence of a systemically diseased population and a role for the microbiome beyond direct oxalate handling in CaOx kidney stone formation. Notably, we identified reduced diversity, altered taxonomic structure, and functional bioenergetic collapse coupled with an enrichment of virulence-associated gene markers in both the urinary and gut microbiota. Markers of health such as vitamin production, butyrate biosynthesis, and core beneficial taxa were comparatively displaced by virulence factors, antimicrobial resistance elements, and pathobionts, in both the urinary and gut microbiota of SF. These multi-site microbial community shifts may be the result of deleterious environmental factors including antibiotic exposure. Based on these findings, we suggest that the historic emphasis put on *O. formigenes* and other direct oxalate-handling taxa should be discontinued in favour of mechanistic study into the apparent systems-level microbial imbalances in SF.

Previous studies investigating the bacterial contribution to this disease, even those employing whole shotgun metagenomic sequencing techniques, have limited their focus to intestinal bacteria that degrade oxalate and the corresponding direct oxalate-handling genes [13, 18, 21, 22, 96, 97]. Several more recent studies have evaluated the gut microbiota in stone disease and described generalized “dysbiosis” and reduced diversity, but a consensus on the specific alterations has not been obtained [16, 17, 22–26]. Moreover, studies into the urinary and stone microbiota of stone formers have been prefatory, involving few patients or lacking an appropriately matched healthy control comparison group [16, 29–32]. Despite lacking a consensus on specific findings, as a collective this previous work has robustly indicated that the microbiome in stone formers significantly differs from a healthy state [98].

It is estimated that 20–50% of urinary oxalate results from dietary consumption [99]. In concordance with previous studies, it was confirmed here that stone patients had higher urinary oxalate concentrations (Fig. 1B) despite comparable measures of dietary oxalate consumption as measured by diet history questionnaire (Fig. 1A) [100]. The urinary microbiota was significantly distinct between HC and SF, and in SF throughout their course of treatment. Specifically, at the time of surgery SF were enriched in inflammatory, antibiotic-resistant, nosocomial infection-associated microbes and their accompanying virulence factors (*Acinetobacter* [101] and *Stenotrophomonas* spp [102]) and compositionally depleted in typically benign members of the urinary microbiota (*Lactobacillus*, *Gardnerella*, and *Prevotella* spp., Fig. 3C) [103]. However, the OR urine samples were collected via catheter (in comparison to the pre-operative samples which were clean-catch), which in addition to the concurrent antibiotic exposure may bias these results. Although further research is needed to conclusively determine the cause, it is possible that relative depletion of the normal urinary microbiota with pre-surgical antibiotic treatment may allow for antibiotic-resistant, deep-seated uropathogens to dominate the niche [104]. Efforts should be made to understand the permanence and clinical implications for such substantial adverse urinary microbiota changes occurring in the short time frame prior to stone surgery.

We confirmed the presence of a sequence-positive microbiota in all stone crystalline compositions, expanding upon previous work by others [16, 29–31, 105]. The stone microbiota was derived from urogenital microbes, yet compositionally distinct from both pre-operative and intraoperative urine (Fig. 2C), indicating that bacteria inside calculi likely do not derive out of coincidental entrapment in the growing stone matrix. Instead, the results suggest that specific microbes are intimately involved in stone development, potentially exacerbating crystal nidus formation through inflammation and crystal aggregation [15, 30, 106, 107]. By fragmenting the calculi, in combination with the often-accompanying antibiotic exposure, surgical stone intervention may be a seeding opportunity for antibiotic-resistant stone-bound bacteria, and perhaps could play a role in the extremely high recurrence rate following surgical stone treatment [108, 109].

The distant site of the oral cavity microbiota was investigated here due to its known associations with systemic diseases such as rheumatoid arthritis [110], cardiovascular disease [111, 112], and cancer [113, 114]. Although not reaching statistical significance with multiple testing corrections, the trends in the current study of increased relative abundance of pathobionts such as *F. nucleatum*, *P. endodontalis*, and *Prevotella* spp. in SF relative to HC further points to a systemically diseased population, rather than the loss of a single gut microbe or function (oxalate degradation). With regard to microbiota analysis, saliva samples are thought to represent the pool from several distinct sites in the oral cavity, so more targeted sampling such as within the periodontal pocket may yield a higher correlation with stone formation. These microbes can easily translocate into circulation through inflamed oral mucosa leading to systemic immune dysregulation [114] and they are also consumed in saliva, seeding the gut microbiota [115]. Thus, it is possible but yet unknown whether the oral microbiota could be acting as a biomarker of KSD or actively implicated in KSD progression; future work should investigate these mechanistic relationships in greater depth.

Longstanding dogma has held that intestinal colonization by *O. formigenes* lowers oxaluria and consequently the risk of stone formation; however, recent studies have failed to detect differences in the colonization rates between stone formers and non-formers [12, 18, 116–118]. In congruence with other recent studies [17, 19, 22], we did not detect a difference in the relative abundance of this bacterium in fecal samples from HC and calcium oxalate (CaOx) SF by whole shotgun metagenomic sequencing. Rather, we found that the gut microbiota significantly differed between cohorts based on alpha diversity, relative taxonomic composition, functional potential, and overall network structure. These alterations are still likely implicated in CaOx nephrolithiasis; however, a clear oxalate-degrading role is not apparent based on these data.

*F. prausnitzii* is a beneficial, butyrate-producing core gut commensal and was found in the current Canadian KiSMi cohort to be significantly depleted in SF in terms of taxonomic abundance as well as through attributed function and viral phage [90, 119]. These findings echo preliminary work by Ticinesi et al. [18] and Liu et al. [26], who performed shotgun metagenomic sequencing on a small number of their Italian and Chinese patients (both *N* = 5 per cohort), as well as Kim et al. [20] and Chen et al. [120] who performed 16S amplicon sequencing on larger Korean and Chinese cohorts, respectively, demonstrating the reproducibility of depleted *Faecalibacterium* in stone formers on a global scale. Although generally associated with health, depletion of this microbe may specifically be involved in nephrolithiasis through its crucial role as a butyrate producer [121]. Butyrate enhances tight junction assembly [122], preventing intestinal permeability and potentially decreasing passive paracellular oxalate uptake [123]. It may also have a role in active transcellular uptake mechanisms by modulating the expression of the oxalate transporter SLC26A6 [124], and even decreasing crystal formation in the kidney through immune modulation [125].

Other gut microbiota alterations detected here have potential implications for nephrolithiasis in SF, including the enrichment of *Desulfovibrio* spp. (a significant signature of SF by *selbal* analysis) and *Flavonifractor plautii*. The intestinal oxalate-sulfate antiporter (SAT-1) has been implicated in human CaOx nephrolithiasis [126]; as sulfate-reducing bacteria, *Desulfovibrio* spp. may reduce the bioavailability of the influx substrate leading to greater plasma oxalate levels, as is observed in cohorts with Autism [127, 128]. *F. plautii* is a flavonoid-degrading bacterium, but dietary plant flavonoids have been shown to be beneficial in stone and cancer cohorts [129], potentially reducing the incidence of CaOx stones through diuretic, anti-oxidant, and anti-inflammatory mechanisms [130]. More broadly, *Eggerthella*, *Flavonifractor*, and *Ruminococcus* spp., all of which were enriched in SF, have recently been suggested as general disease-associated signatures (shared across type 2 diabetes, diarrhoea and constipation, mental disorders, and gallstones) in a cohort of over 8000 extensively characterized Dutch individuals [90]. Additionally, the SF gut was enriched in a variety of uropathogenic Gammaproteobacteria including *Pseudomonas* spp. and *Proteus* spp. (significant signatures of SF by *selbal* analysis) as well as *Klebsiella* spp.. The gut is a known reservoir for uropathogens [131], thus the relevance of these organisms’ enrichment in SF goes beyond intestinal colonization as these bacteria have themselves been cultured from calcium-based stones, and may be exacerbating formation [31, 106]. The balance analysis indicating *Desulfovibrio piger*, *Proteus*, and *Pseudomonas* spp. as significant signatures of SF relative to unclassified taxa from the phyla Bacillota and Fusobacteriota requires further confirmatory study, but these taxa hold promise as potential future biomarkers of stone disease diagnosis or pathogenesis.

Previous literature demonstrates that antibiotic exposure is associated with stone formation [21, 132]. In congruence, our findings that SF had significantly more recent antibiotic use, and the enrichment in antibiotic resistance genes and taxa support the notion that antibiotic exposure (from childhood up to and during stone treatment) corrupts both the urinary and gut microbiota leading to inflammation, loss of beneficial function, and a bloom of uropathogens which may be exacerbating stone formation [133].

Although this work has demonstrated a critical association of the microbiota in kidney stone disease, limitations of the current study must be considered. These data were derived from a single centre from a relatively ethnically homogenous patient population, and thus should be replicated in an independent and ethnically diverse cohort. Several patient factors including smoking status, UTI, and antibiotic exposure history were discordant between the current cohorts, so a future study with larger matched sample sizes would increase the precision of the current findings (particularly the predicted biomarker taxa). No differences were determined between the HC and SF cohorts based on diet as measured by the validated food frequency questionnaire; however, results from this questionnaire may be biased through self-reporting and yearly estimation. Future studies could employ a standardized diet or detailed food diary to more accurately estimate the dietary micro- and macronutrients directly prior to sample collection. In a similar manner, the participants’ antibiotic exposure history was self-reported, which is also subject to bias. In future studies where the healthcare infrastructure permits, a more detailed medical record of antibiotic exposures should be utilized.

This study employed 16S rRNA amplicon sequencing of the urinary and saliva samples, and whole shotgun metagenomic sequencing of the stool samples; these techniques provide relative compositional, but not absolute abundance information. Both sequencing methods did not provide taxonomic annotation to the species level in all cases. For this reason, as well as the problematic nature of comparing across sequencing methodologies, caution should be taken when comparing these data and taxonomic annotations in future studies. Further, future research could employ genomic assemblies from the shotgun dataset to explore species-specific taxonomy. The 16S rRNA amplicon data were used to infer functional metagenomes with PICRUSt2; although this technique demonstrates accuracy in samples of human origin, it is based on variation in the amplicon region to predict the entire genome, and thus is unable to differentiate strain-level functionality [47, 48]. This technique is not a replacement for whole shotgun metagenomic sequencing, which could be employed in the future to validate the current findings. Although fecal samples were collected in accordance with a previously validated protocol, they were collected on toilet paper and may have been exposed to skin-derived or environmental microbes. HC and initial SF urine samples were collected as clean-catch midstream samples; however, this may not capture bacteria adhered to the uroepithelium. Rather than the clean-catch method, SF-OR samples were collected during surgery via catheter, which likely accounts for some of the differences between the SF urine samples over time; in the future, HC and initial SF urine samples could also be collected via catheter for additional comparability. Although a multivariate analysis was undertaken, a future study in a larger sample size could have better precision in detecting and adjusting for potential confounders due to patient comorbidities and lifestyle factors. Finally, this was an observational study, thus we cannot attribute causality of stone disease to the differences detected between the microbiota of HC and SF.

## Conclusions

Together, the SF-associated markers detected in this study are indicative of a systemically diseased population and may hold greater explanatory power regarding nephrolithiasis risk than direct oxalate degradation or *O. formigenes* presence. We propose that a more inconspicuous method of oxalate homeostasis is likely causative and dependent on an evolutionarily adapted gut microbiota. If the diversity and robust functional potential of the healthy human microbiome is repeatedly assaulted by the average Westernized lifestyle via antibiotic exposure, diet, and other environmental factors, kidney stone prevalence will continue to increase. To prevent the cyclic recurrence of this disease, future management of nephrolithiasis should incorporate both the prevention of microbiome disturbance and the use of agents for subsequent restoration of homeostasis.

### Supplementary Information

**Additional file 1. **Supplementary Data.

**Additional file 2: Supplementary Figure 1.** KiSMi cohort sample collection. ** Supplementary Figure 2. **Dietary macronutrients are comparable between cohorts.** Supplementary Figure 3. **Kidney stone microbiota is not dictated by stone composition.** Supplementary Figure 4. **Oral salivary microbiota is comparable between cohorts.** Supplementary Figure 5. **Gut microbiota global balances predictive of kidney stone disease.** Supplementary Table 1. **KiSMi study participant inclusion and exclusion criteria.** Supplementary Table 2. 1**6S rRNA primer and barcode sequences. **Supplementary Table 3. **HPLC running conditions.

## Data Availability

The datasets supporting the conclusions of this article are available in the NCBI Sequence Read Archive: BioProject ID PRJNA856314 (urinary 16S dataset), and PRJNA856641 (oral 16S dataset), and PRJNA649273 (gut shotgun metagenomic dataset). Input counts, taxonomy annotation, and metadata tables are available at Zenodo (https://doi.org/10.5281/zenodo.7199455), and custom scripts are available at https://github.com/kait-al/KiSMi-Kidney-Stone-Microbiome. All other data generated or analysed during this study are included in this published article and its supplementary information files.

## Abbreviations

BMI: Body mass index
CaOx: Calcium oxalate
CLR: Centred log ratio
FDR: False discovery rate
GLM: Generalized linear model
HC: Healthy controls
HPLC: High-performance liquid chromatography
IBD: Inflammatory bowel disease
KSD: Kidney stone disease
OR: Operating room
PCA: Principal component analysis
PSNL: Percutaneous nephrolithotomy
SF: Stone formers
SV: Sequence variant
URS: Ureteroscopy
UTI: Urinary tract infection
