# Supplementary material for: Multi-site microbiota alteration is a hallmark of kidney stone formation
Source: Microbiome. 2023 Nov 25;11:263. doi: 10.1186/s40168-023-01703-x (PMC10675928; doi:10.1186/s40168-023-01703-x)
Supplement: Supplementary file 3 — Additional file 2: Supplementary Figure 1. KiSMi cohort sample collection. Supplementary Figure 2. Dietary macronutrients are comparable between cohorts. Supplementary Figure 3. Kidney stone microbiota is not dictated by stone composition. Supplementary Figure 4. Oral salivary microbiota is comparable between cohorts. Supplementary Figure 5. Gut microbiota global balances predictive of kidney stone disease. Supplementary Table 1. KiSMi study participant inclusion and exclusion criteria. Supplementary Table 2. 16S rRNA primer and barcode sequences. Supplementary Table 3. HPLC running conditions. [file 40168_2023_1703_MOESM2_ESM.pdf]

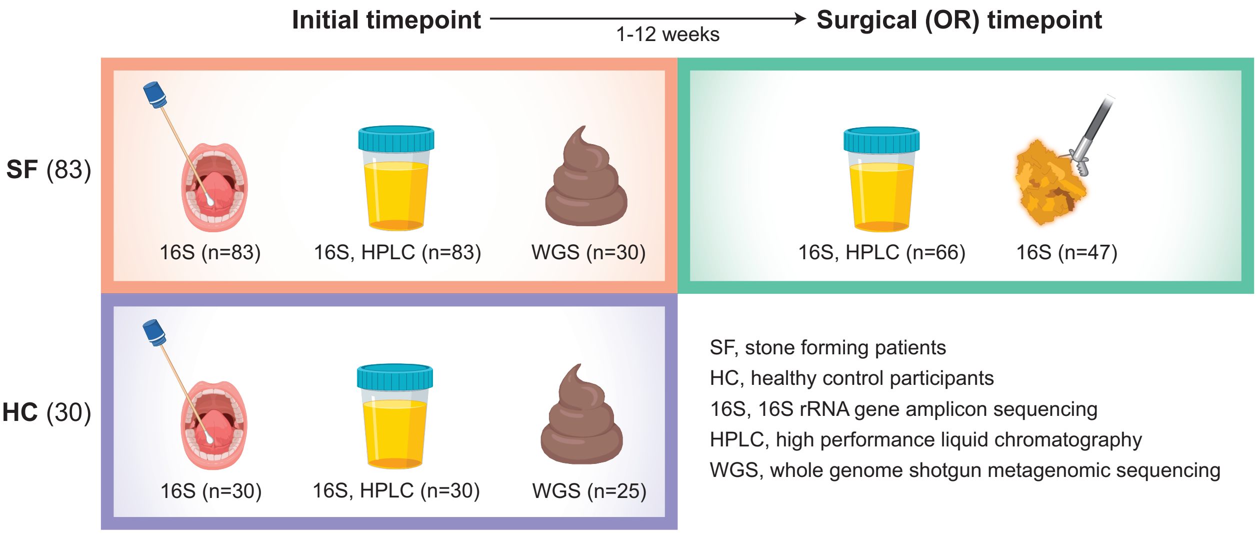

Supplementary Figure 1. KiSMi cohort sample collection. Sublingual saliva, midstream urine, and fecal samples were collected from SF and HC prior to antibiotic exposure (within the last 30 days); additional urine and stone fragments were collected from SF during stone removal surgery.

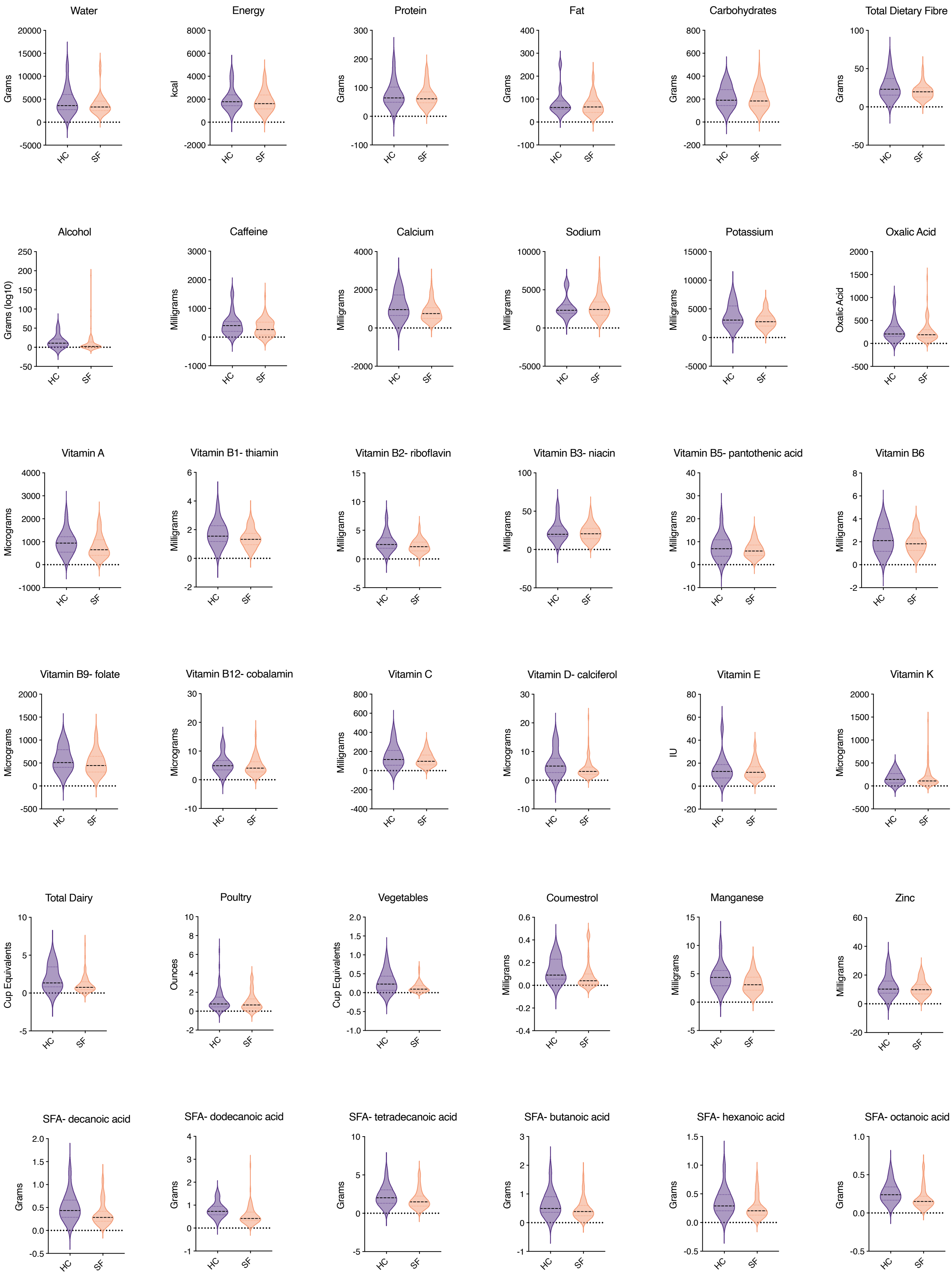

Water
Grams
Energy
kcal
Protein
Grams
Fat
Grams
Carbohydrates
Grams
Total Dietary Fibre
Grams
Alcohol
Grams (log10)
Caffeine
Milligrams
Calcium
Milligrams
Sodium
Milligrams
Potassium
Milligrams
Oxalic Acid
Oxalic Acid
Vitamin A
Micrograms
Vitamin B1- thiamin
Milligrams
Vitamin B2- riboflavin
Milligrams
Vitamin B3- niacin
Milligrams
Vitamin B5- pantothenic acid
Milligrams
Vitamin B6
Milligrams
Vitamin B9- folate
Micrograms
Vitamin B12- cobalamin
Micrograms
Vitamin C
Milligrams
Vitamin D- calciferol
Micrograms
Vitamin E
IU
Vitamin K
Micrograms
Total Dairy
Cup Equivalents
Poultry
Ounces
Vegetables
Cup Equivalents
Coumestrol
Milligrams
Manganese
Milligrams
Zinc
Milligrams
SFA- decanoic acid
Grams
SFA- dodecanoic acid
Grams
SFA- tetradecanoic acid
Grams
SFA- butanoic acid
Grams
SFA- hexanoic acid
Grams
SFA- octanoic acid
Grams
HC
SF

Supplementary Figure 2. Dietary macronutrients are comparable between cohorts

Approximate daily values of macronutrients were compared between patient groups; no macronutrients were significantly different by two-tailed Mann Whitney test after multiple testing corrections. HC = healthy control participants (n = 14); SF = stone former (n = 64). Violin plots represent the data distribution, with dashed lines representing the median and quartiles. Select dietary values are shown, see Supplementary Data 1B–C for full output.

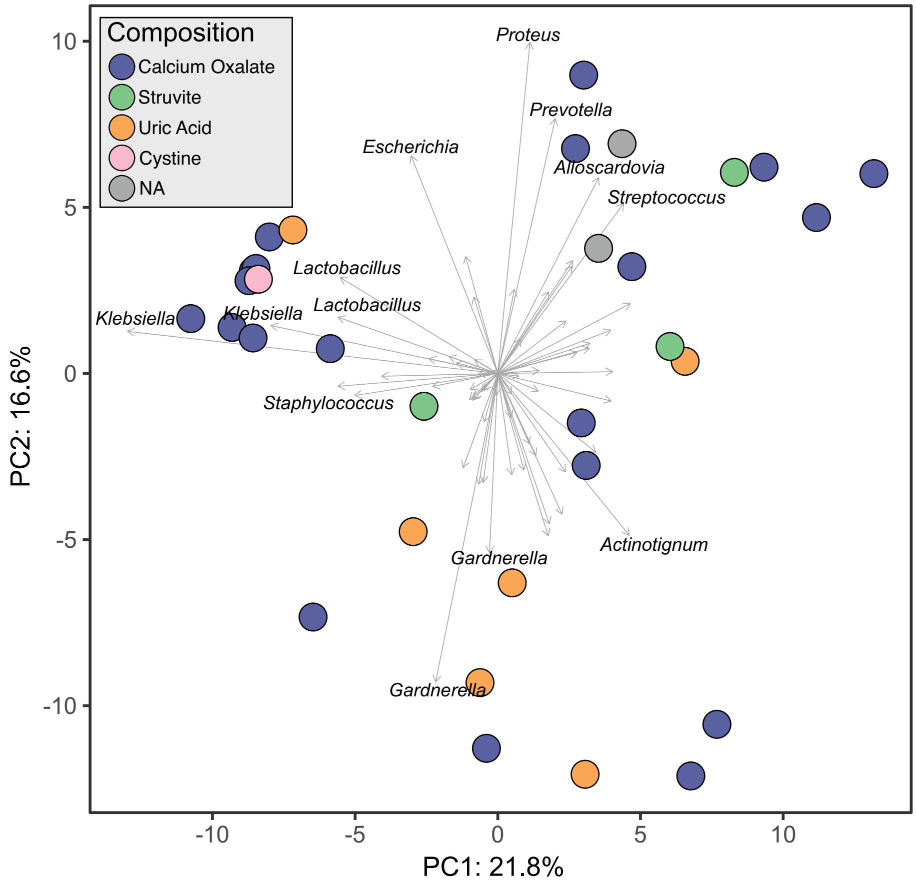

Supplementary Figure 3. Kidney stone microbiota is not dictated by stone composition

PCA was performed on CLR-transformed Aitchison distances of all stone samples. Each coloured point represents a sample. Distance between samples on the plot represents differences in microbial community composition, with 38.4% of total variance being explained by the first two components shown. Strength and association for genera are depicted by the length and direction of the gray arrows, respectively. Samples are coloured by stone composition (major component shown for stones with mixed composition). NA = stone composition is not available as fragments were not sent for analysis.

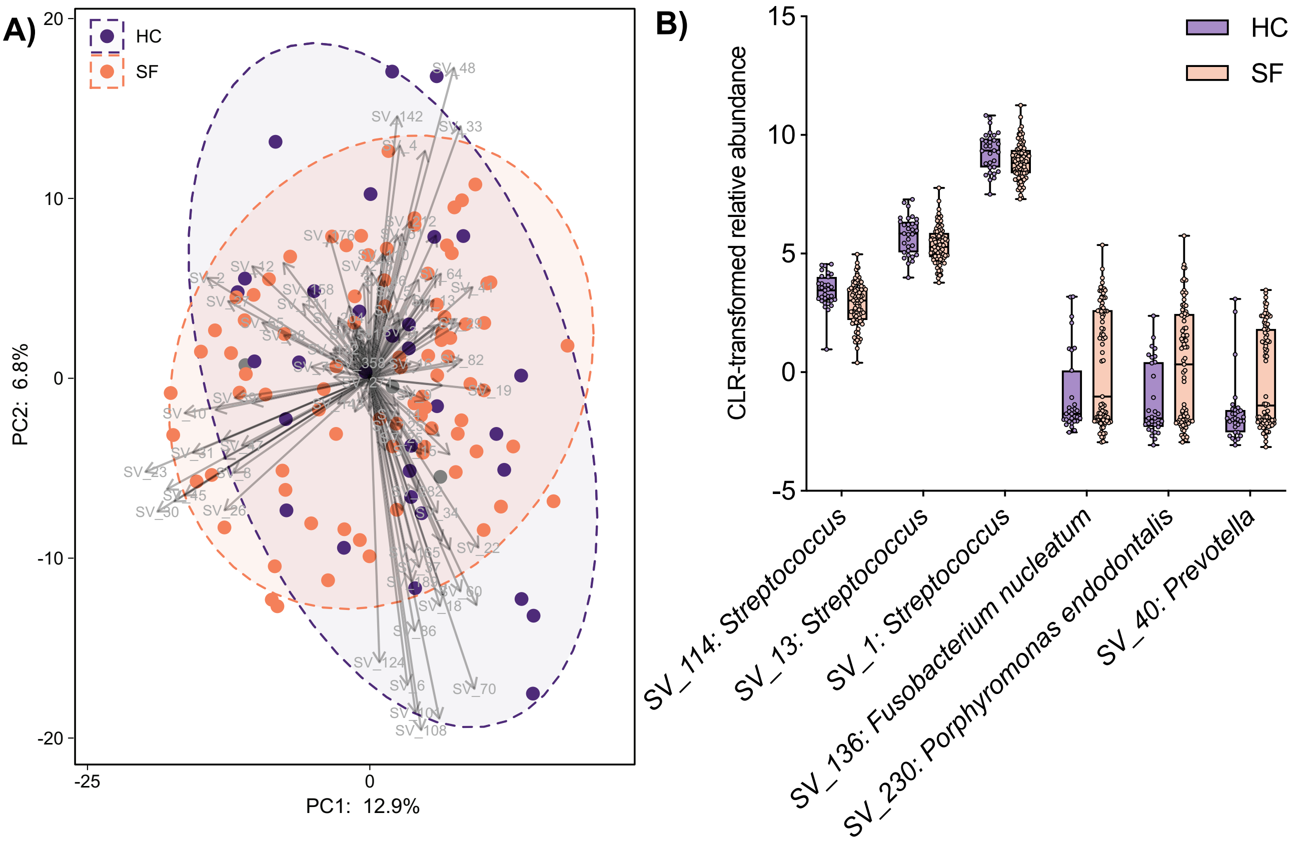

Supplementary Figure 4. Oral salivary microbiota is comparable between cohorts

A) PCA was performed on CLR-transformed Aitchison distances of all oral saliva samples. Each coloured point represents a sample. Distance between samples on the plot represents differences in microbial community composition, with 19.7% of total variance being explained by the first two components shown. Strength and association for genera are depicted by the length and direction of the gray arrows, respectively. Samples are coloured by cohort. **B)** Most divergent SVs between SF and HC, stone former OR urine are displayed, although none were statistically significant after multiple testing correction. Data represent the median, IQR, and range; HC (n = 30), SF (n = 83).

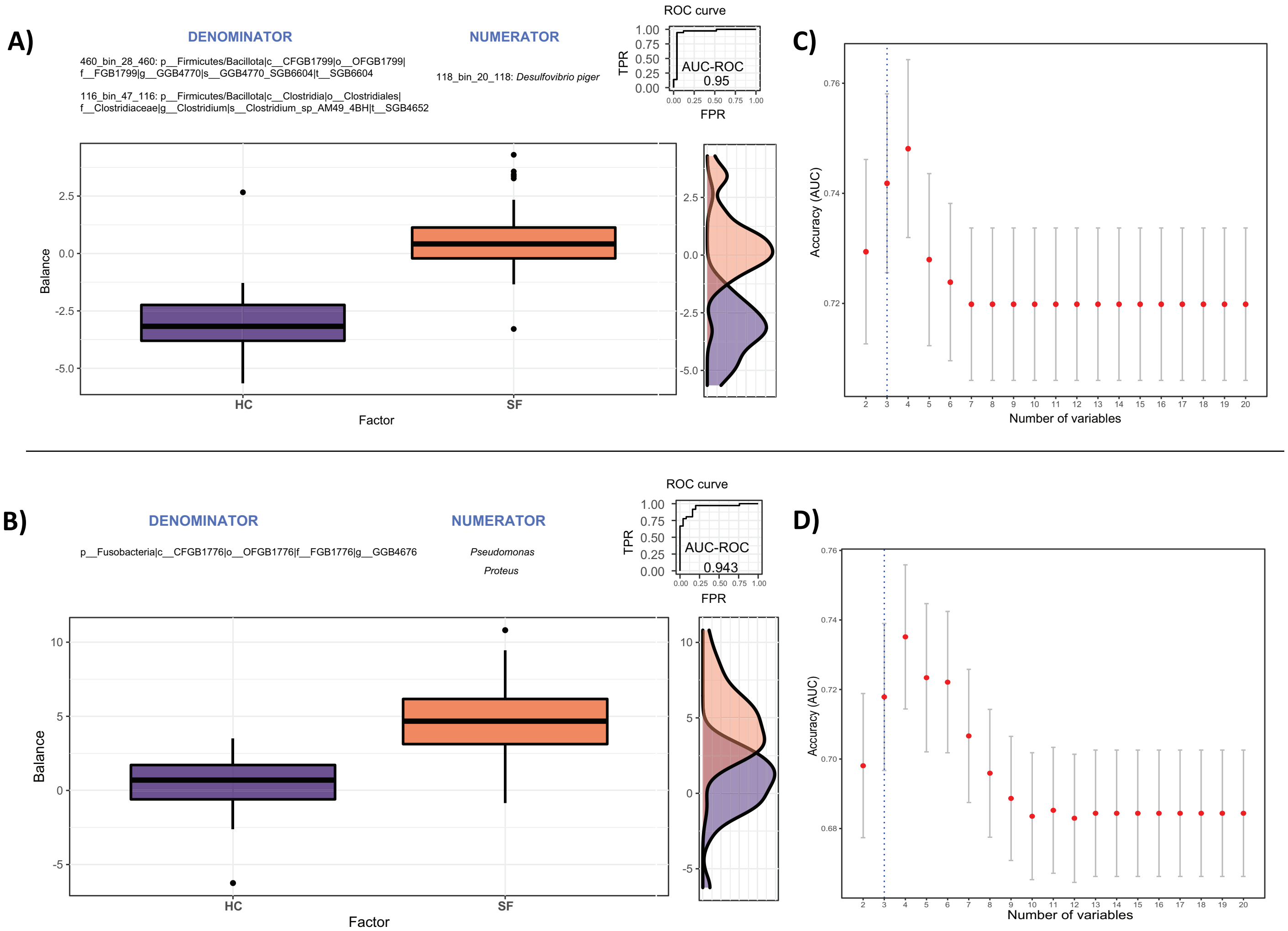

Supplementary Figure 5. Gut microbiota global balances predictive of kidney stone disease

A) and B) display the global plots of taxa and genera respectively whose balance most discriminates between HC (n = 25) and SF (n = 35). Both the boxplots and the density curves reveal that HC have lower balance scores than SF, meaning lower relative abundances of those taxa in the numerator with respect to those in the denominator. The discrimination values (AUC-ROC) are 0.95 for taxonomic bins and 0.943 for genera balances. C) and D) display the mean area under the receiver operating characteristic (ROC) curve (AUC) as a function of the number of components included in the balance in the cross-validation process. The optimal number of components according to the "1st rule" is 3 for both the taxonomic bin and genera global balances. The mean cross-validated AUC was 0.7418 for the taxonomic bin balance, and 0.7179 for genera balance. Analysis was performed in accordance with Rivera-Pinto et al. (2018).

Supplementary Table 1. KiSMi study participant inclusion and exclusion criteria

**Kidney stone patients**	
**Inclusion**	**Exclusion**
At least 18 years if age	Taking/taken any antibiotic during the previous 30 days
Scheduled for PCNL or URS as treatment of urinary stone disease	Has an active gastro-intestinal infection
Able and willing to provide informed consent	Previously enrolled in this trial
Able and willing to provide urine, saliva, and stool samples	In the opinion of the treating urologist, it is not in the patient's best interest to participate
Able and willing to complete diet questionnaire at home	
**Healthy control participants**	
**Inclusion**	**Exclusion**
At least 18 years if age	Taking/taken any antibiotic during the previous 30 days
Able and willing to provide informed consent	Has an active gastro-intestinal infection
Able and willing to provide urine, saliva, and stool samples	Has Crohn's disease
Able and willing to complete diet questionnaire at home	Has ulcerative colitis
Undergo renal ultrasound to confirm stone-free status	Has had gastric bypass surgery
	Has a history of urinary stone disease
	Has a history of urosepsis in the past year (365 days)
	Has a urinary diversion
	Has an indwelling catheter
	Performs clean intermittent catheterization
	Has previously enrolled in this trial
	In the opinion of the treating urologist, it is not in the subject's best interest to participate

Supplementary Table 2. 16S rRNA primer and barcode sequences

Primer	Sequence (5'-3')
Golay_L1	ACACTCTTTCCCTACACGACGCTCTTCCGATCTNNNNTGCATACACTGGGTGCCAGCMGCCGCGGTAA
Golay_L2	ACACTCTTTCCCTACACGACGCTCTTCCGATCTNNNNACTCACAGGAATGTGCCAGCMGCCGCGGTAA
Golay_L3	ACACTCTTTCCCTACACGACGCTCTTCCGATCTNNNNGTAGGTGCTTACGTGCCAGCMGCCGCGGTAA
Golay_L4	ACACTCTTTCCCTACACGACGCTCTTCCGATCTNNNNCAGTCGTTAAGAGTGCCAGCMGCCGCGGTAA
Golay_L5	ACACTCTTTCCCTACACGACGCTCTTCCGATCTNNNNCACTACGCTAGAGTGCCAGCMGCCGCGGTAA
Golay_L6	ACACTCTTTCCCTACACGACGCTCTTCCGATCTNNNNGCTCGAAGATTCGTGCCAGCMGCCGCGGTAA
Golay_L7	ACACTCTTTCCCTACACGACGCTCTTCCGATCTNNNNTGAACGTTGGATGTGCCAGCMGCCGCGGTAA
Golay_L8	ACACTCTTTCCCTACACGACGCTCTTCCGATCTNNNNATGGTTCACCCGGTGCCAGCMGCCGCGGTAA
Golay_L9	ACACTCTTTCCCTACACGACGCTCTTCCGATCTNNNNCGAGGGAAAGTCGTGCCAGCMGCCGCGGTAA
Golay_L10	ACACTCTTTCCCTACACGACGCTCTTCCGATCTNNNNTACTACGTGGCCGTGCCAGCMGCCGCGGTAA
Golay_L11	ACACTCTTTCCCTACACGACGCTCTTCCGATCTNNNNGTTCCTCCATTAGTGCCAGCMGCCGCGGTAA
Golay_L12	ACACTCTTTCCCTACACGACGCTCTTCCGATCTNNNNACGATATGGTCAGTGCCAGCMGCCGCGGTAA
Golay_L13	ACACTCTTTCCCTACACGACGCTCTTCCGATCTNNNNTATCGACACAAGGTGCCAGCMGCCGCGGTAA
Golay_L14	ACACTCTTTCCCTACACGACGCTCTTCCGATCTNNNNAGCATGTCCCGTGTGCCAGCMGCCGCGGTAA
Golay_L15	ACACTCTTTCCCTACACGACGCTCTTCCGATCTNNNNCCAGATATAGCAGTGCCAGCMGCCGCGGTAA
Golay_L16	ACACTCTTTCCCTACACGACGCTCTTCCGATCTNNNNGTGTCCGGATTCGTGCCAGCMGCCGCGGTAA
Golay_L17	ACACTCTTTCCCTACACGACGCTCTTCCGATCTNNNNATCGCACAGTAAGTGCCAGCMGCCGCGGTAA
Golay_L18	ACACTCTTTCCCTACACGACGCTCTTCCGATCTNNNNCAGCTCATCAGCGTGCCAGCMGCCGCGGTAA
Golay_L19	ACACTCTTTCCCTACACGACGCTCTTCCGATCTNNNNGCATATGCACTGGTGCCAGCMGCCGCGGTAA
Golay_L20	ACACTCTTTCCCTACACGACGCTCTTCCGATCTNNNNTGTAGGTGTGCTGTGCCAGCMGCCGCGGTAA
Golay_L21	ACACTCTTTCCCTACACGACGCTCTTCCGATCTNNNNACGAGACTGATTGTGCCAGCMGCCGCGGTAA
Golay_L22	ACACTCTTTCCCTACACGACGCTCTTCCGATCTNNNNCATCAGTACGCCGTGCCAGCMGCCGCGGTAA

Golay_<br>L23	ACACTCTTTCCCTACACGACGCTCTTCCGATCTNNNNGTATCTGCGCGTG<br>TGCCAGCMGCCGCGGTAA
Golay_<br>L24	ACACTCTTTCCCTACACGACGCTCTTCCGATCTNNNNTGCGTCAGCTACG<br>TGCCAGCMGCCGCGGTAA
Golay_<br>R1	CGGTCTCGGCATTCCTGCTGAACCGCTCTTCCGATCTNNNNCGAGGGAA<br>AGTCGGACTACHVGGGTWTCTAAT
Golay_<br>R2	CGGTCTCGGCATTCCTGCTGAACCGCTCTTCCGATCTNNNNTACTACGTG<br>GCCGGACTACHVGGGTWTCTAAT
Golay_<br>R3	CGGTCTCGGCATTCCTGCTGAACCGCTCTTCCGATCTNNNNGTTCCTCCA<br>TTAGGACTACHVGGGTWTCTAAT
Golay_<br>R4	CGGTCTCGGCATTCCTGCTGAACCGCTCTTCCGATCTNNNNACGATATGG<br>TCAGGACTACHVGGGTWTCTAAT
Golay_<br>R5	CGGTCTCGGCATTCCTGCTGAACCGCTCTTCCGATCTNNNNTATCGACAC<br>AAGGGACTACHVGGGTWTCTAAT
Golay_<br>R6	CGGTCTCGGCATTCCTGCTGAACCGCTCTTCCGATCTNNNNAGCATGTCC<br>CGTGGACTACHVGGGTWTCTAAT
Golay_<br>R7	CGGTCTCGGCATTCCTGCTGAACCGCTCTTCCGATCTNNNNCCAGATATA<br>GCAGGACTACHVGGGTWTCTAAT
Golay_<br>R8	CGGTCTCGGCATTCCTGCTGAACCGCTCTTCCGATCTNNNNGTGTCCGGA<br>TTCGGACTACHVGGGTWTCTAAT
Golay_<br>R9	CGGTCTCGGCATTCCTGCTGAACCGCTCTTCCGATCTNNNNATCGCACAG<br>TAAGGACTACHVGGGTWTCTAAT
Golay_<br>R10	CGGTCTCGGCATTCCTGCTGAACCGCTCTTCCGATCTNNNNCAGCTCATC<br>AGCGGACTACHVGGGTWTCTAAT
Golay_<br>R11	CGGTCTCGGCATTCCTGCTGAACCGCTCTTCCGATCTNNNNGCATATGCA<br>CTGGGACTACHVGGGTWTCTAAT
Golay_<br>R12	CGGTCTCGGCATTCCTGCTGAACCGCTCTTCCGATCTNNNNTGTAGGTGT<br>GCTGGACTACHVGGGTWTCTAAT
Golay_<br>R13	CGGTCTCGGCATTCCTGCTGAACCGCTCTTCCGATCTNNNNACGAGACTG<br>ATTGGACTACHVGGGTWTCTAAT
Golay_<br>R14	CGGTCTCGGCATTCCTGCTGAACCGCTCTTCCGATCTNNNNCATCAGTAC<br>GCCGGACTACHVGGGTWTCTAAT
Golay_<br>R15	CGGTCTCGGCATTCCTGCTGAACCGCTCTTCCGATCTNNNNGTATCTGCG<br>CGTGGACTACHVGGGTWTCTAAT
Golay_<br>R16	CGGTCTCGGCATTCCTGCTGAACCGCTCTTCCGATCTNNNNTGCGTCAGC<br>TACGGACTACHVGGGTWTCTAAT
Golay_<br>R17	CGGTCTCGGCATTCCTGCTGAACCGCTCTTCCGATCTNNNNGTAGATCGT<br>GTAGGACTACHVGGGTWTCTAAT
Golay_<br>R18	CGGTCTCGGCATTCCTGCTGAACCGCTCTTCCGATCTNNNNCAGCTGGTT<br>CAAGGACTACHVGGGTWTCTAAT
Golay_<br>R19	CGGTCTCGGCATTCCTGCTGAACCGCTCTTCCGATCTNNNNAGCTGATAG<br>TTGGGACTACHVGGGTWTCTAAT
Golay_<br>R20	CGGTCTCGGCATTCCTGCTGAACCGCTCTTCCGATCTNNNNTCTACGGCA<br>CGTGGACTACHVGGGTWTCTAAT
Golay_<br>R21	CGGTCTCGGCATTCCTGCTGAACCGCTCTTCCGATCTNNNNGCATAAACG<br>ACTGGACTACHVGGGTWTCTAAT

Golay_R22	CGGTCTCGGCATTCCTGCTGAACCGCTCTTCCGATCTNNNNAAGGCGCTCCTTGGACTACHVGGGTWTCTAAT
Golay_R23	CGGTCTCGGCATTCCTGCTGAACCGCTCTTCCGATCTNNNNTGCCTAAGATCGGGACTACHVGGGTWTCTAAT
Golay_R24	CGGTCTCGGCATTCCTGCTGAACCGCTCTTCCGATCTNNNNCTTAGCTACTCTGGACTACHVGGGTWTCTAAT

Supplementary Table 3. HPLC running conditions

	Creatinine quantification	Oxalate quantification
**Flow rate**	1 mL/minute	1 mL/minute
**Mobile phase**	60% ACN / 40% 5 mM $KHPO_4$ (pH 2.8)	15% ACN / 85% 50 mM $KHPO_4$ (pH 7.0)
**Column**	Poroshel 120 HILIC (4.6 × 150 mm, 4μm) – ambient temperature (Agilent)	Poroshel 120 EC-C18 (4.6 × 150 mm, 4μm) – ambient temperature (Agilent)
**Injection volume**	5 μL	5 μL
**Detector**	215, 4 (ref = 320, 120)	312. 4 (ref = 355, 50)
**Retention time**	~2.2 minutes	~ 2.7 minutes
**Run time**	4 minutes	4 minutes
